# CXCL16/CXCR6 interaction promotes endometrial decidualization via the PI3K/AKT pathway

**DOI:** 10.1530/REP-18-0417

**Published:** 2019-01-08

**Authors:** Jie Mei, Yuan Yan, Shi-Yuan Li, Wen-Jie Zhou, Qun Zhang, Ming-Qing Li, Hai-Xiang Sun

**Affiliations:** 1 Reproductive Medicine Center, Department of Obstetrics and Gynecology, Nanjing Drum Tower Hospital, The Affiliated Hospital of Nanjing University Medical School, Nanjing, Jiangsu, China; 2 Nanjing University Medical School, Nanjing, Jiangsu, China; 3 Laboratory for Reproductive Immunology, Key Laboratory of Reproduction Regulation of NPFPC, SIPPR, IRD, Hospital of Obstetrics & Gynecology, Fudan University, Shanghai, China; 4 Department of Obstetrics and Gynecology, Nanjing Drum Tower Hospital, The Affiliated Hospital of Nanjing University Medical School, Nanjing, Jiangsu, China

## Abstract

Decidualization renders the endometrium transiently receptive to an implanting blastocyst although the underlying mechanisms remain incompletely understood. The aim of this study was to determine the role of chemokine CXCL16 and its receptor CXCR6 in the decidualization during pregnancy. Here, the expression of CXCL16 was investigated in endometrial tissues, decidua and placenta in this study. Compared with endometrial tissue, protein expression of CXCL16 was significantly higher in tissues from the fertile control samples, especially in villus. Meanwhile, the primary trophoblast cells and decidual stromal cells (DSCs) secreted more CXCL16 and expressed higher CXCR6 compared to endometrial stromal cells (ESCs)* in vitro*. Stimulation with the inducer of decidualization (8-bromoadenosine 3′,5′-cyclic with medroxyprogesterone acetate, 8-Br-cAMP plus MPA) significantly upregulated the expression of CXCL16 and CXCR6 in ESCs *in vitro*. After treatment with exogenous recombinant human CXCL16 (rhCXCL16) or trophoblast-secreted CXLC16, decidualised ESCs showed a significant decidual response, mainly characterised by increased prolactin (PRL) secretion. Simultaneously, PI3K/PDK1/AKT/Cyclin D1 pathway in decidualised ESCs were activated by rhCXCL16, and AKT inhibitor GS 690693 abolished the PRL secretion of ESCs that was triggered by rhCXCL16. Finally, the impaired CXCL16/CXCR6 expression could be observed at the maternal–foetal interface from patients who have experienced spontaneous abortion. This study suggests that the CXCL16/CXCR6 axis contributes to the progression of ESC decidualization by activating PI3K/PDK1/AKT/Cyclin D1 pathway. It unveils a new paradigm at the maternal–foetal interface in which CXCL16 is an initiator for the molecular crosstalk that enhances decidualization of ESCs.

## Introduction

For successful pregnancy, the human endometrium must first engage with a competent embryo, embed the conceptus in decidualising stroma and then support deep uterine invasion of extraembryonic trophoblast (Wang & Dey 2006). Decidualisation denotes the differentiation process by which resident ESCs acquire a specialised secretory phenotype. It bestows on stromal cells the ability to create paracrine gradients essential for uterine receptivity and post-implantation pregnancy support (Cloke *et al.* 2008). Secreted factors, such as prolactin (PRL) and insulin-like growth factor-binding protein 1 (IGFBP1), are widely used to assess the quality of the decidual response in human endometrial stromal cells (ESCs) (Al-Sabbagh *et al.* 2011). Decidualisation involves coordination of several processes, including polyploidy, and several types of molecules, including cytokine receptors, enzymes, morphogens, hormones and transcription factors. However, the mechanisms that control the decidualisation process over the window of implantation are largely unknown.

Chemokines are a superfamily of chemotactic cytokines, which were initially studied for their role in regulation of leukocyte trafficking to inflammatory sites. The chemokine superfamily consists of nearly 50 cytokine members and 20 chemokine receptors (Stone *et al.* 2017). Recently, it was revealed that chemokines play an essential role in pregnancy support. Abnormal expression of chemokines has been connected with conditions such as recurrent spontaneous abortion, preeclampsia and cancer metastasis (Nancy *et al.* 2012). On one hand, decidual cells surrounding the early conceptus have been shown to epigenetically silence key chemokine genes, thus protecting the allogeneic foetus from attack of cytotoxic lymphocytes (Nancy *et al.* 2012). On the other hand, chemokines (such as interleukin 12) could also induce the proinflammatory microenvironment and underpin endometrial receptivity and early implantation in pregnancy (Hajipour *et al.* 2018). Therefore, the decidual cells could adapt dynamically and tailor their chemokine secretome to accommodate the various stages of the implantation process (Guzeloglu-Kayisli *et al.* 2009). However, the function of chemokine in the process of decidualization remains to be elucidated.

CXCL16 is a member of the plasma membrane chemokines, consisting of a chemokine domain followed by a glycosylated mucin-like stalk, a single transmembrane helix and a short cytoplasmic tail (Di Castro *et al.* 2016). The soluble CXCL16 induces migration in CXCR6-expressing cells, whereas the transmembrane molecule functions as a scavenger receptor for oxidised low-density lipoprotein and an adhesion molecule to CXCR6-expressing cells (Lang & Lang 2015, Yu *et al.* 2016).

Based on the evidence that CXCL16 is impaired at the human maternal–foetal interface in patients who experience recurrent pregnancy loss (RPL) (Huang *et al.* 2008, Fan *et al.* 2018), we hypothesised that CXCL16 secreted by decidual or trophoblast cells might regulate the process of decidualization during pregnancy. Here, we investigate the expression of CXCL16 and its receptors at the human maternal–foetal interface. We further provide the evidence about its effects on decidualization process, as well as its regulatory mechanism.

## Materials and methods

### Subjects and sample collection

The subjects recruited into the study were women of reproductive age attending our department between October 2016 and October 2017. Written informed consent was obtained from all patients before sampling, and all the biopsies were taken solely for research purposes. The study protocol was approved by the Research Ethics Committee of Nanjing Drum Tower Hospital. Control endometrium tissues were collected at hysterectomy from patients with leiomyoma (31 cases, age: 38.2 ± 9.14 years). The secretory phase of the menstrual cycle was determined by the histopathological evaluation. None of the included patients took any medications or received hormonal therapy within 6 months prior to surgery and none had experienced complications related to pelvic inflammatory diseases. Human villus tissues were obtained from 106 women with clinically normal pregnancies (age: 30.13 ± 7.32 years; gestational age at sampling: 49.67 ± 7.84 days (mean ± s.d.)) (termination for nonmedical reasons). Normal decidual tissues were obtained from 25 women with clinically normal pregnancies (age: 28.19 ± 9.23 years; gestational age at sampling: 50.12 ± 9.12 days (mean ± s.d.)) (termination for nonmedical reasons). The spontaneously aborted decidual tissues were collected from 21 patients who experienced an embryonic gestation (age: 31.97 ± 6.11 years; gestational age at sampling: 56.36 ± 9.8 days (mean ± s.d.)). Their ultrasound results showed an intrauterine embryo without cardiac activity at more than 7.5–8 weeks of gestation. Uterine curettage is performed when the pregnancy has been confirmed to be nonviable. Pregnancy was confirmed by ultrasound and blood tests, and the women who had spontaneous abortion because of endocrine, anatomical and genetic abnormalities, as well as infection, were excluded. All samples of at least 300 mg were collected under sterile conditions and divided into two parts. The first part was immediately fixed in 4% v/v paraformaldehyde for immunohistochemistry studies, and the second part was transported to the laboratory on ice in Dulbecco modified Eagle medium (DMEM)/F-12 medium (Gibco) within 30 min after surgery.

### CXCL16 localization by immunohistochemistry

Immunohistochemistry was performed on specimens of human normal endometrium, and decidual and villus tissues from normal pregnancies or spontaneous abortion. Paraffin sections were cut at a thickness of 5 mm. Slides were dewaxed, hydrated and quenched in 3% hydrogen peroxide. They were then soaked in 0.01 M citric acid (pH 6.0) for 20 min at 95°C for antigen retrieval. After cooling, slides were blocked for 60 min using 1% bovine serum albumin (BSA; Hyclone) in Tris buffered saline. The samples were then incubated with goat anti-human polyclonal CXCL16 antibody (10 μg/mL, R&D) at 4°C overnight in a humidity chamber. As a negative control, the primary antibody was replaced with isotype-matched immunoglobulin G (IgG) (Sino-America Co. Ltd, Shanghai, China). Two washings with PBS were performed before the slides were incubated with the secondary antibody (80 ng/mL, biotinylated anti-goat, Santa Cruz Biotechnology). The slides were developed in peroxidase substrate solution (Vector Laboratories) for 3.5 min, counterstained with haematoxylin for 10 min and then washed in running tap water for 30 min. Finally, the slides were evaluated under Olympus BX51 + DP70 microscope (Olympus Optical). Densitometry of the immunohistochemistry was assessed with ImageJ software (W.S. Rasband, National Institutes of Health, Bethesda, MD, USA).

### RNA isolation and quantitative real-time PCR

Total RNA was extracted from human normal endometrium and decidual and villus tissues from normal pregnancies using a TRIzol reagent (Invitrogen) according to the manufacturer’s instructions. One microgram of total RNA was reverse transcribed in a total volume of 20 μL. Reverse transcription was performed using random primers, and quantitative real-time polymerase chain reaction (qRT-PCR) was conducted with a MyiQ Single-Color Real-Time PCR Detection System (Bio-Rad). The following primers were also used for *cxcl16 and actin*: 5′-GCCATCGGTTCAGTTCA-3′ and 5′-CAATCCCCGAGTAAGCAT-3′ for *cxcl16: 5*′*- CCTCTATGCCAACACAGT -3*′,* 5*′*- AGCCACCAATCCACACAG-3*′* for actin*. Reactions were run in duplicate using RNA samples. The fold change in expression of each gene was calculated using the 2^−△△CT^ method, with actin as an internal control.

### Isolation of human ESCs, DSCs and trophoblasts

The endometrium tissue, decidua and villus tissues were washed in Hank balanced salt solution. We purified ESCs as described previously (Li *et al.* 2012). The isolation of human trophoblasts from villus tissues and DSCs from decidua were both treated according to a previously described method (Guo *et al.* 2010). This method supplies a 95% purity of vimentin−cytokeratin (CK)7+ trophoblast cells and greater than 98% vimentin+CK7− ESCs and DSCs, which was confirmed by FCM analysis.

### *In vitro* decidualization of ESCs

ESCs decidualization was induced by the addition of 8-bromoadenosine 3′,5′-cyclic adenosine monophosphate (8-Br-cAMP, 0.5 μM) with medroxyprogesterone acetate (MPA, 10^−6^ M) for 9 days and measured by insulin-like growth factor-binding protein-1 (IGFBP-1) and prolactin (PRL) level in the culture supernatants according to the method described in the study by Klemmt *et al.* (Klemmt *et al.* 2006). Normal ESCs were seeded into 24-well plates (2 × 10^5^ cells/well) and treated with phenol red-free DMEM/F-12 medium containing 2% dextran-coated charcoal-treated foetal bovine serum (FBS, Gibco) in the presence of 8-Br-cAMP (0.5 μM; Sigma) and MPA (10^−6^ M; Sigma) for 9 days. DSCs were also cultured as the positive control for 9 days. The medium was changed every 3 days. Culture supernatants (1, 3, 6 and 9 days) were collected and centrifuged to remove cellular debris and stored at −80°C for IGFBP-1 and PRL measurements.

### Cell culture and treatment of ESCs

To evaluate the expression of CXCL16/CXCR6 in ESCs, DSCs or trophoblast cells, ESCs and DSCs were seeded into 24-well plates (2 × 10^5^ cells/well) and incubated in DMEM/F-12 medium containing 10% v/v FBS; trophoblast cells were in DMEM medium (Hyclone) containing 20% v/v FBS. The medium was changed every 3 days. The cells (1, 3, 6 and 9 days) were collected for flow cytometry. The culture supernatants (1, 3, 6, and 9 days) were also collected and centrifuged to remove cellular debris and stored at −80°C for IGFBP-1 and PRL measurements.

In addition, to clarify the effect of CXCL16 on ESCs decidualization, decidualised ESCs (ESCs incubated with cAMP plus MPA for 6 days) were then treated with 100 ng/mL recombinant human CXCL16 (rhCXCL16; Cell Signaling Technology), or 5 µg/mL CXCL16 antagonist (anti-CXCL16; Cell Signaling Technology) or the culture supernatants of trophoblast cells, or with culture media of trophoblast (high-glucose DMEM containing 20% v/v FBS), or with vehicle (0.1% v/v dimethyl sulfoxide; Sigma) as control for 3 days. Additionally, the PI3K-AKT signalling pathway inhibitor 10 µM GSK 690693 (Sigma) was added in the past 24 h.

### Measurement of CXCL16, IGFBP-1 and PRL by ELISA

The secretion of CXCL16, IGFBP-1 and PRL in the cultured supernatant samples was determined by enzyme-linked immunosorbent assay (ELISA) using commercially available kits. They were detected with the CXCL16 ELISA Kits (R&D), IGFBP-1 ELISA Kits (R&D) and PRL ELISA Kits (R&D) according to the manufacturer’s protocol. The absorbance value was measured by a DigiScan Microplate Reader. Cultured cells were homogenised, and the total protein in the homogenised cells was measured by a bicinchoninic acid protein assay kit (Pierce). Data were standardised by total protein of cell lysates.

### Measurement of CXCR6 by flow cytometry

The endometrium and decidual tissues from normal pregnancies or spontaneous abortion were minced into 2- to 3-mm pieces and digested with collagenase type IV (0.1%; Sigma) and deoxyribonuclease type I (DNase I; 3000 U; Sigma) with constant agitation for 70 min at 37°C. Then, the resulting cell suspension, as well as the cultured ESCs, DSCs or trophoblast cells, were stained with PE anti-CXCR6 monoclonal antibody (2.5 μL; Biolegend, CA, USA). The cells were concurrently stained with anti-IgG2a-PE as controls. After the addition of the monoclonal antibodies, the samples were allowed to react at 4°C for 30 min. Then, the cells were suspended in PBS and centrifuged at 400 ***g*** for 8 min. The cells were resuspended in 0.5 mL PBS and were analysed by FacsCalibur BD flow cytometry (Becton Dickinson). The resulting data were analysed using the LYSYS II software programme (Becton Dickinson).

### CXCL16 measurement and PI3K/AKT pathway detection by western blotting

To quantitate the protein expression of CXCL16 in maternal–foetal interface, the endometrium and decidual and villus tissues from normal pregnancies or spontaneous abortion were minced into 2- to 3-mm pieces, washed in PBS (Hyclone), and centrifuged for 15 min at 2000 ***g*** at 4°C for the preparation of tissue lysates. To validate the signalling pathway responsible for decidualization induced by CXCL16, we investigated the effect of rhCXCL16 on the PI3K-AKT phosphorylation. Decidualised ESCs (treated with cAMP + MPA for 6 days) were incubated with rhCXCL16, and cell lysates were obtained at each of the indicated times up to 60 min (0, 10, 30, and 60 min) by detachment with the cell scraper. Cell lysates prepared for western blotting tissue and cell pellets were resuspended in high-efficiency cell tissue rapid lysis buffer (radioimmunoprecipitation assay; Beyotime) containing 1% v/v phenylmethanesulfonylfluoride (Beyotime) proteinase and 1% w/v phosphatase inhibitors (Roche, USA). Cell lysates were heated for 5 min at 95°C and then stored at −80°C. Protein concentrations were quantified using the protein assay kit (Beyotime). Total proteins (40 mg) were electrophoresed in 10% sodium dodecyl sulphate-polyacrylamide gels (Beyotime) on a Miniprotean III system (Bio-Rad) and blotted onto 0.45 mm PVDF membrane (Millipore, MA, USA) for 2 h at 48°C. The membranes were then blocked with 5% w/v BSA for 2 h, and further incubated with antibodies against rabbit anti-human CXCL16 polyclonal (1:1000, Product NO. #ab101404; Abcam), mouse anti-human PI3K (p85) monoclonal antibody (0.18 μg/mL, Cell Signaling Technology), rabbit anti-human Phospho-PI3K (p85) antibody (1:100, Product NO. #4228, Cell Signaling Technology), rabbit anti-human Phospho-AKT (Ser473) (0.9 μg/mL, Cell Signaling Technology), AKT (0.35 μg/mL, Cell Signaling Technology), Cyclin D1 (0.85 μg/mL, Cell Signaling Technology) at 4°C overnight. The blots were then washed and incubated at room temperature for 1 h in peroxidase-conjugated goat anti-mouse or goat anti-rabbit IgG secondary antibodies (80 ng/mL; Santa Cruz Biotechnology). The blots were washed five times and then processed for chemiluminescence with a SuperSignal West Duraextended duration substrate kit (Thermo Fisher Scientific, IL, USA).

### Statistical analysis

Data were analysed by one-way analysis of variance and least significant difference (equal variances assumed), or Tamhane test (equal variances not assumed) was used *post hoc* for multiple comparisons. All the analysis was performed using GraphPad Prism 6 software (GraphPad Software, Inc.). A value of *P* < 0.05 was considered to indicate a statistically significant difference.

## Results

### CXCL16/CXCR6 is elevated at the maternal–foetal interface during decidualization

**Figure 1:**
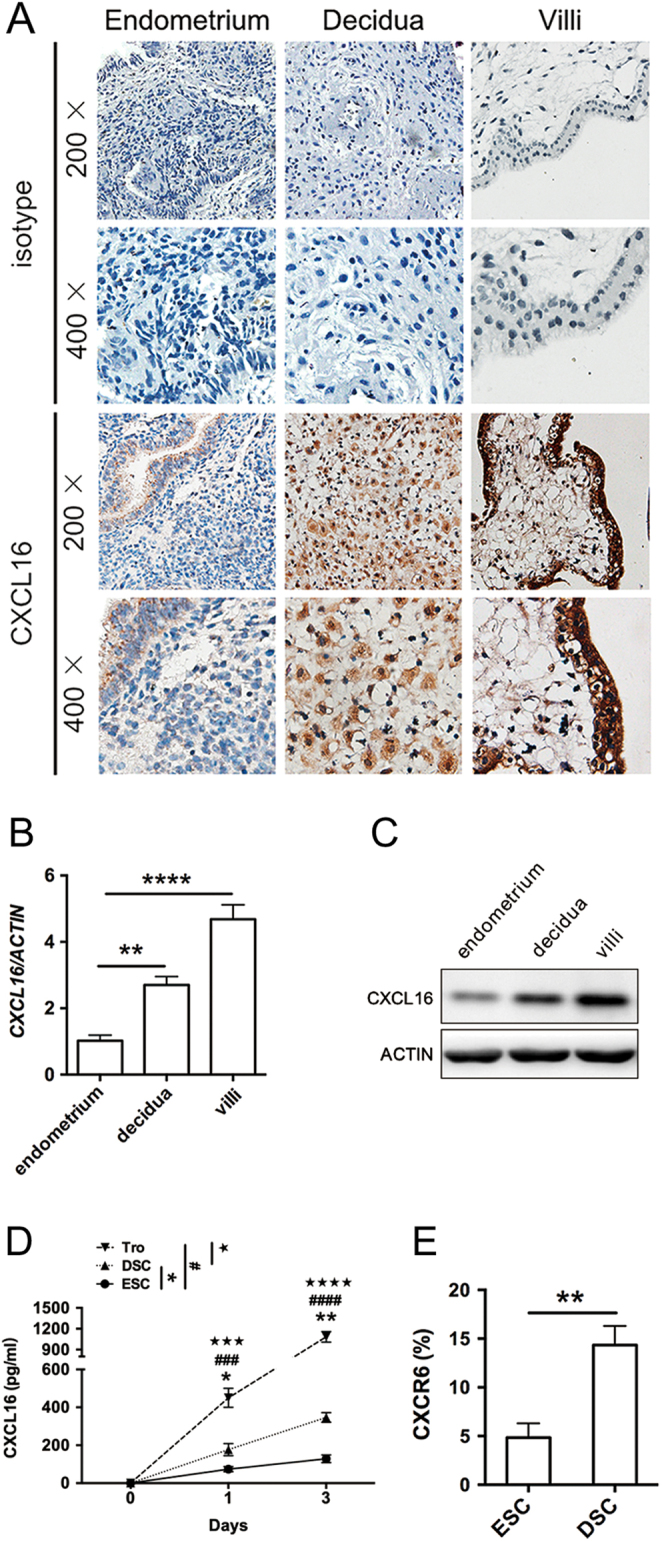
Expression of CXCL16/CXCR6 at the maternal–foetal interface during decidualization. (A) Representative micrographs for CXCL16 immunostaining in normal endometrium from a control group (ii), decidual tissues (iii), and villus (iv) from normal pregnancies. Negative antibody isotype staining of the normal tissue was represented as control (i). Magnification, ×200, ×400. (B and C) The CXCL16 transcript and translation levels in endometrium, decidual tissues and villus were quantified by real-time PCR and immunoblotting and were then normalised to actin expression. (D) To identify the secretory CXCL16 level of ESCs, DSCs and trophoblast cells, the culture supernatants (1 and 3 days) were collected for CXCL16 measurements by ELISA. (E) The expression of CXCR6 in DSCs and trophoblast cells was detected in 3 days by flow cytometry. Decidua, decidua from normal pregnancy; DSC, decidual stromal cell from normal pregnancy; endometrium, normal endometrium; ESC, endometrial stromal cell; Tro, trophoblast cells from normal pregnancy; villi, villus tissues from normal pregnancy. Representative data were shown as well as the mean ± s.d. (*n* = 12). Scale bar = 50 mm. **P < 0.01, ****P < 0.0001. In (C), DSC vs ESC, *P < 0.05, **P < 0.01; Tro vs ESC, ^###^*P* < 0.001, ^####^*P* < 0.0001; Tro vs DSC, ^★★★^*P* < 0.001, ^★★★★^*P* < 0.0001.

To investigate whether CXCL16/CXCR6 is expressed at the maternal–foetal interface, we performed immunohistochemistry, RT-PCR, immunoblotting, ELISA, and FCM to detect the CXCL16/CXCR6 level in the endometrial or pregnancy tissue from control samples. As shown in Fig. 1A and B, CXCL16 was expressed in normal endometrium and maternal–foetal interface. Its transcript and translation levels were elevated in decidual tissues from normal pregnancies than endometrium. In addition, a markedly higher level of CXCL16 could also be observed in villus tissues. Next, we evaluated CXCL16/CXCR6 expression in ESCs, DSCs, and trophoblast cells by ELISA and FCM. The freshly isolated ESCs, DSCs, and trophoblast cells were cultured for 1 and 3 days. We found that DSCs supernatant secreted a 3.1-fold CXCL16 than ESCs on the third day (*P* < 0.01, Fig. 1C), and trophoblast cells released even higher level of CXCL16 than DSCs in 3 days (*P* < 0.001, Fig. 1C). Similar to the CXCL16 secretion, CXCR6 was higher in DSCs than ESCs as shown in Fig. 1D.

**Figure 2:**
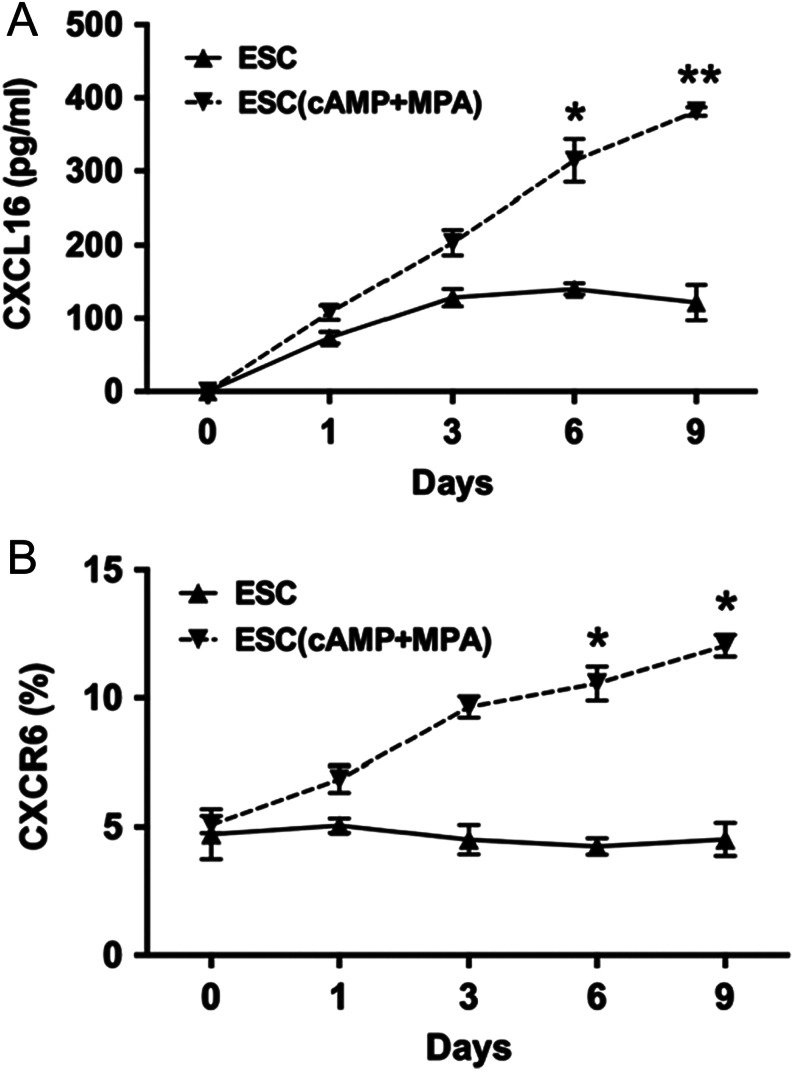
CXCL16 and CXCR6 expression in *in vitro* decidualised ESCs. ESCs and decidualised ESCs (ESCs treated with cAMP plus MPA) were incubated (1, 3, 6, 9 days) for CXCL16 secretion (A) by ELISA and CXCR6 expression (B) by flow cytometry. Data are expressed as the mean ± s.d. (*n* = 12). ESC, endometrial stromal cell; ESC (cAMP + MPA), *in vitro* decidualised ESCs, ESCs incubated with MPA plus cAMP. ESC(cAMP + MPA) vs ESC, *P < 0.05, **P < 0.01. (vs ESC group in 6 and 9 days).

Next, we analysed the expression of CXCL16/CXCR6 in *in vitro* decidualised ESCs (ESCs incubated with cAMP plus MPA for 9 days). During 9 days of cell culture, in comparison with ESCs, the *in vitro* decidualised ESCs (ESC (cAMP + MPA)) secreted more soluble CXCL16 and expressed higher level of CXCR6 from the sixth day (*P* < 0.05, Fig. 2A and B).

### Trophoblast-derived CXCL16 accelerates ESC decidualization *in vitro*

**Figure 3:**
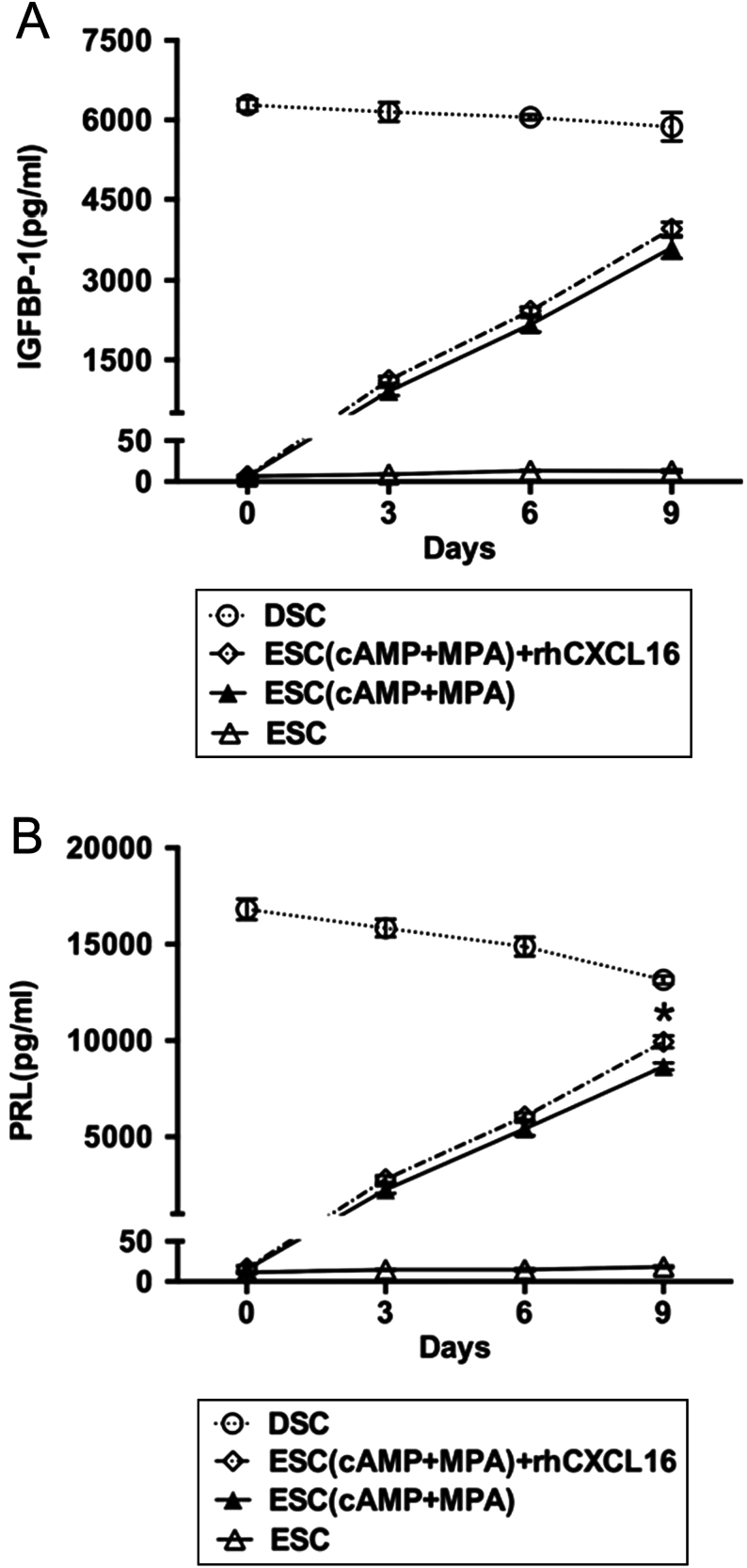
Effect of exogenous CXCL16 on ESC decidualization *in vitro*. ESCs were treated with rhCXCL16 (100 ng/mL, 3 days) following an *in vitro* decidualization protocol. The culture supernatants (1, 3, 6 and 9 days) were collected and centrifuged to remove cellular debris, and for IGFBP-1 (A), PRL (B) measurements. DSC, decidual stromal cell from normal pregnancy; ESC (cAMP + MPA) + rhCXCL16, *in vitro* decidualised ESCs treated with rhCXCL16; ESC (cAMP + MPA), *in vitro* decidualised ESCs, ESCs incubated with MPA plus cAMP; ESC, endometrial stromal cell. Data are expressed as the mean ± s.d. (*n* = 12). ESC(cAMP + MPA) + rhCXCL16 vs ESC(cAMP + MPA), *P < 0.05.

**Figure 4:**
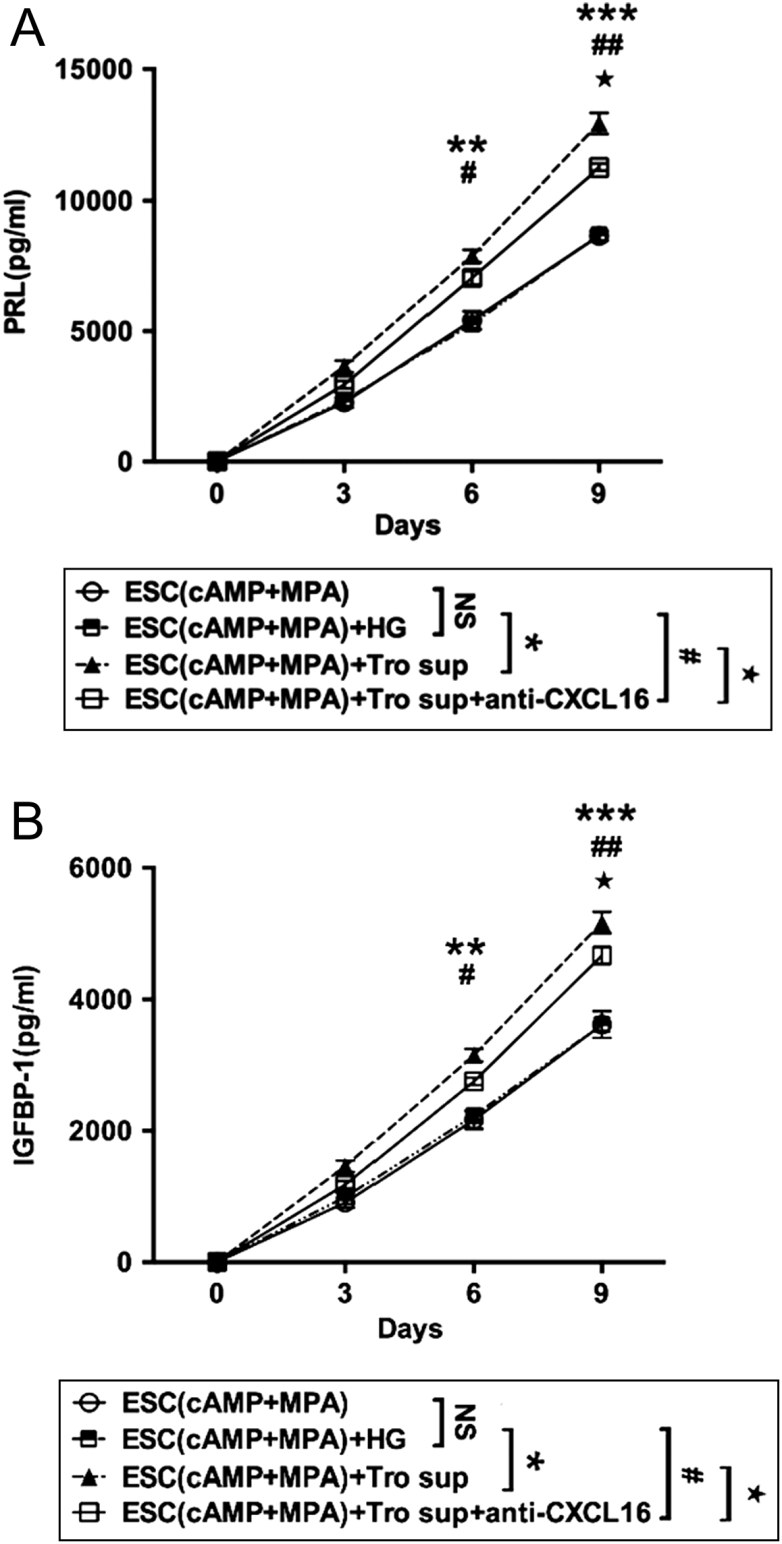
Role of trophoblast-derived CXCL16 on ESC decidualization *in vitro*. The *in vitro* decidualised ESCs were treated with high-glucose DMEM (HG), culture supernatants of trophoblast cells or anti-CXCL16 (5 µg/mL) for 3 days. Decidualised ESCs treated with trophoblast cells supernatants secreted higher IGFBP-1 (A) and PRL (B) in 6 and 9 days (ESCs(cAMP + MPA)+ Tro sup vs ESCs(cAMP + MPA) + HG, **P < 0.01), but the secretion level reduced when added with anti-CXCL16 in 9 days (ESCs(cAMP + MPA)+ Tro sup vs ESCs(cAMP + MPA)+ Tro sup+anti-CXCL16, ^★^*P* < 0.05). ESC (cAMP + MPA) + HG, *in vitro* decidualised ESCs treated with high-glucose DMEM containing 20% v/v FBS; ESC (cAMP + MPA) + Tro sup, *in vitro* decidualised ESCs treated with supernatants of trophoblast cells; ESC (cAMP + MPA) + Tro sup+anti-CXCL16, *in vitro* decidualised ESCs treated with supernatants of trophoblast cells and anti-CXCL16; ESC (cAMP + MPA), *in vitro* decidualised ESCs, ESCs incubated with MPA plus cAMP. Data are expressed as the mean ± s.d. (*n* = 12). ESCs(cAMP + MPA)+Tro sup vs ESCs(cAMP + MPA)+ HG, **P < 0.01, ***P < 0.001; ESCs(cAMP + MPA) +Tro sup+anti-CXCL16 vs ESCs(cAMP + MPA) + HG, ^#^*P* < 0.05, ^##^*P* < 0.01; ESCs(cAMP + MPA) +Tro sup+anti-CXCL16 vs ESCs(cAMP + MPA) +Tro sup, ^★^*P* < 0.05.

To further evaluate the effect of CXCL16 on ESC decidualization, we treated *in vitro* decidualised ESCs with exogenous rhCXCL16 for 3 days, and found that rhCXCL16 could elevate the secretion of PRL in decidualised ESC on the ninth day (*P* < 0.05, Fig. 3B), but not IGFBP-1 (*P* > 0.05, Fig. 3A). Because the trophoblast cells secreted a high level of CXCL16, we treated decidualised ESCs with the supernatant of trophoblast cells. From the sixth day, significantly higher IGFBP-1 and PRL could be detected in decidualised ESCs (*P* < 0.01, Fig. 4A and B), which could be partly reduced after anti-CXCL16 administration (*P* < 0.05, Fig. 4A and B). This suggested that culture supernatants of trophoblast cells could promote the decidualization by other factors except CXCL16.

### CXCL16 promotes ESC decidualization via PI3K-PDK1-AKT-Cyclin D1 signalling pathway

**Figure 5:**
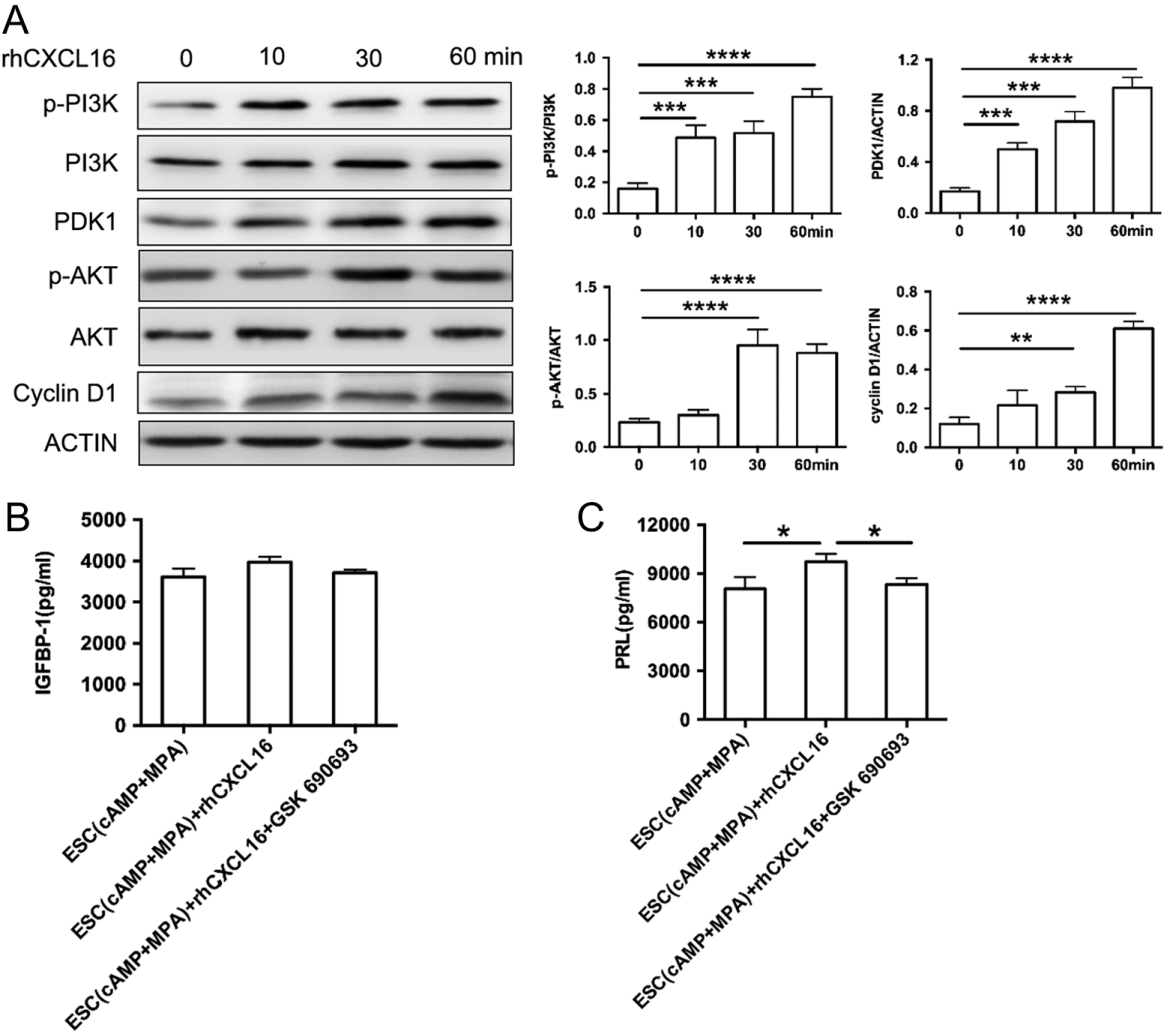
Signalling pathway activated by CXCL16 in ESCs decidualization. (A) *In vitro*-decidualised ESCs were incubated to 100 ng/mL rhCXCL16 and cell lysates were obtained at each of the indicated times up to 60 min (0, 10, 30, 60 min). Lysates were prepared for immunoblotting by using p-PI3K/PI3K, p-AKT/AKT and PDK1, Cyclin D1 antibodies (*left panels*), and the ratio of protein to actin (*right panels)* are shown. *In vitro* decidualised ESCs were treated with rhCXCL16, either in the presence or absence of the AKT signalling pathway inhibitor 10 µM GSK 690693 (10 µM, 24 h). The culture supernatants were collected for the detection of IGFBP-1 (B) and PRL (C) secretion. ESC (cAMP + MPA) + rhCXCL16 + GSK 690693, *in vitro* decidualised ESCs treated with rhCXCL16 and GSK 690693; ESC (cAMP + MPA) +rhCXCL16, *in vitro*-decidualised ESCs treated with rhCXCL16; ESC (cAMP + MPA), *in vitro*-decidualised ESCs, ESCs incubated with MPA plus cAMP. Data are expressed as the mean ± s.d. (*n* = 6). *P < 0.05.

To validate the intracellular signalling mechanisms responsible for decidualization induced by CXCL16, we investigated the effect of CXCL16 on the PI3K-AKT pathways. We found that rhCXCL16 induced the phosphorylation of PI3K after a 10-min incubation, and AKT was also significantly phosphorylated 30 min later. Meanwhile, the downstream PDK1 and cyclin D1 were elevated 10 and 60 min later respectively (Fig. 5A). In addition, our studies also indicated that rhCXCL16-treated *in vitro* decidualised ESCs exhibited significantly lower PRL expression after treatment of GSK 690693 (AKT inhibitor) (*P* < 0.05, Fig. 5C). There were no significant differences of IGFBP-1 secretion in decidualised ESCs treated with rhCXCL16 or GSK 690693 (*P* > 0.05, Fig. 5B). The result verified the rapid and highly efficient effect of rhCXCL16 on PI3K-AKT signalling pathway.

### Impaired CXCL16/CXCR6 expression at the maternal–foetal interface in patients experiencing spontaneous abortion

**Figure 6:**
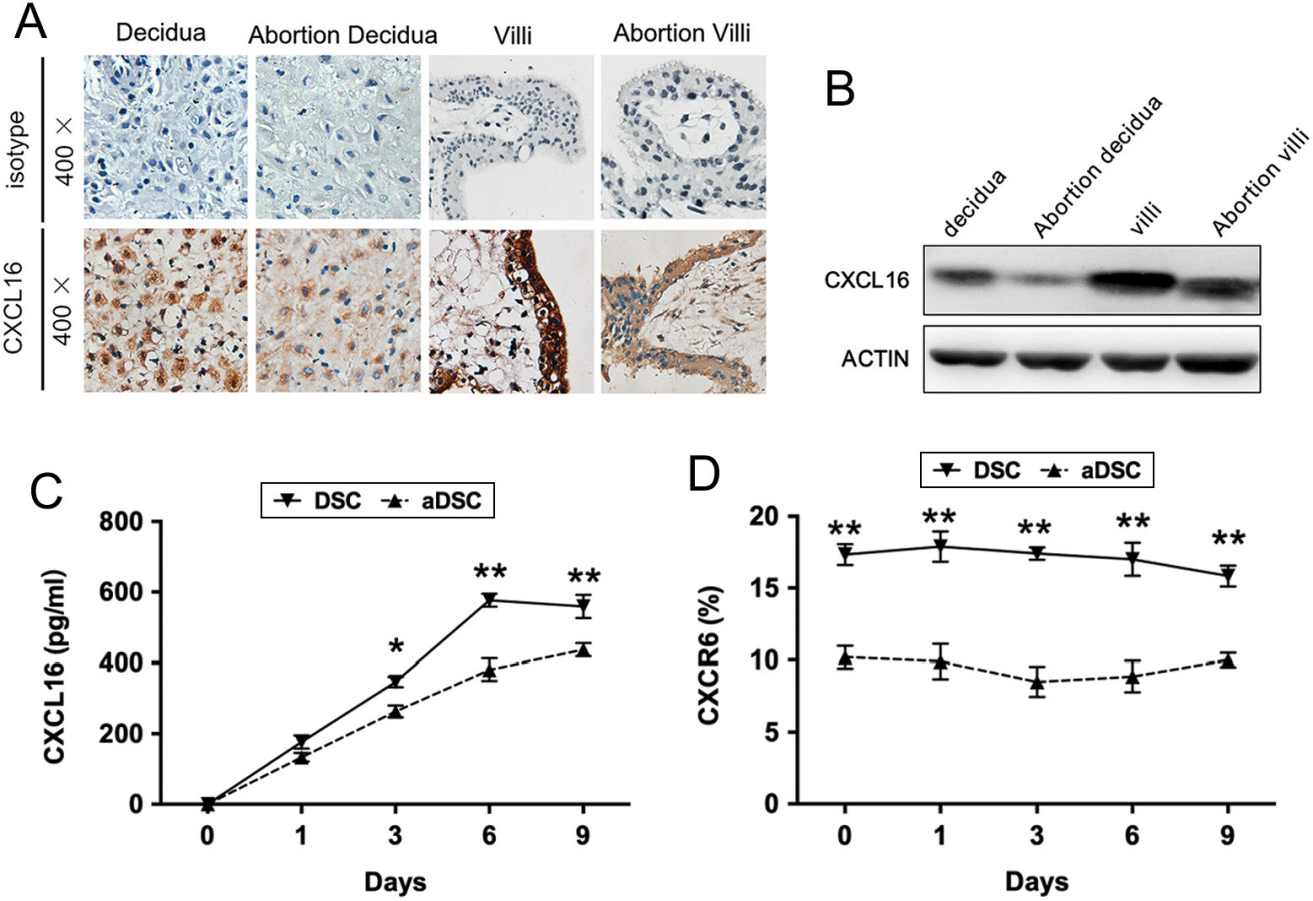
CXCL16/CXCR6 expression at the maternal–foetal interface in patients experiencing spontaneous abortion. (A) Representative micrographs for CXCL16 immunostaining in decidual tissues from normal pregnancies (i) and patients who experienced spontaneous abortion (ii), villus tissues from normal pregnancies (iii) and patients who experienced spontaneous abortion (iv), respectively. Magnification, ×200, ×400. (B) The protein level of CXCL16 in decidual tissues and villus from normal pregnancies and patients who experienced spontaneous abortions were quantified by immunoblotting. (C) The culture supernatants (1, 3, 6 and 9 days) of DSCs and aDSCs were collected for CXCL16 measurements by ELISA. (D) CXCR6 expression of DSCs from patients who experienced spontaneous abortion was determined by flow cytometry in different time points (1, 3, 6 and 9 days). Abortion decidua, decidua from spontaneous abortion patients; abortion villi, villus tissues from spontaneous abortion patients; aDSC, decidual stromal cell from spontaneous abortion; decidua, decidua from normal pregnancy; DSC, decidual stromal cell from normal pregnancy; villi, villus tissues from normal pregnancy. Representative data were shown as well as the mean ± s.d. of 12 different experiments. Scale bar = 50 mm. aDSC vs DSC, **P < 0.01.

Finally, we compared the CXCL16/CXCR6 expression of decidua and villus from normal pregnancies with the patients who had experienced spontaneous abortion. In immunohistochemistry and immunoblotting, the decreased changes of CXCL16 and CXCR6 were observed in the decidua and villus from patients who had undergone spontaneous abortion. CXCL16 secretion and CXCR6 expression were both reduced in DSCs from patients who had undergone spontaneous abortion (aDSCs) than normal ones after 6 days of cell culture (*P* < 0.01, Fig. 6B and C).

## Discussion

In this study, we investigated the potential role of CXCL16/CXCR6 in promoting decidualization during pregnancy process. We demonstrated that, compared with normal pregnant tissue, CXCL16/CXCR6 was markedly decreased in villus and decidual tissues from patients who had undergone spontaneous abortion. In addition, via PI3K/AKT/Cyclin D1 pathway, CXCL16 secreted by trophoblast cells could contribute to the induction of decidualization *in vitro*.

The development of a successful pregnancy depends on maternal receptivity during the implantation window. It is largely established during decidualization, when the stromal cells of the endometrium undergo structural and morphological changes to prepare for possible embryo implantation (Vinketova *et al.* 2016). Impaired decidualization, measured by a reduction in the decidualization marker prolactin (PRL) in the endometrium, has been associated with recurrent miscarriage (Salker *et al.* 2010). Despite intensive studies of decidualization, its exact mechanism remains unclear.

Chemokines are a large family of chemotactic proteins with low molecular weight. Secretion of appropriate chemokine signals by decidual cells contributes to the recruitment of predominantly anti-inflammatory leukocyte subpopulations necessary for pregnancy maintenance (He *et al.* 2012). Chemokines mediate the generation of the maternal–placental interface through the interaction between DSCs, trophoblast cells and the selective recruitment of leukocytes (Ramhorst *et al.* 2016). CXCL16, as a member of the chemokine family, is expressed and released by numerous types of cells such as dendritic, liver sinusoidal endothelial and cancer cells and macrophages (Lekva *et al.* 2017). CXCL16 has been documented to have diverse effects on the initiation and development of multiple diseases. It can activate T cells and inflammatory molecules, which promote atherosclerosis (Yin *et al.* 2017), as well as participate in the proliferation and migration of cancer cells (Deng *et al.* 2010). To our knowledge, the role of CXCL16 during pregnancy has not been thoroughly evaluated.

As reported in the studies by Huang *et al.*, chemokine CXCL16 was found in the cytoplasm and cytomembrane of human cytotrophoblasts, syncytiotrophoblasts and extravillous trophoblasts in the first trimester. CXCR6 as the sole receptor of CXCL16 is preferentially expressed on decidual cells, mainly in the decidual immune cells (Huang *et al.* 2006, 2008). Fan *et al.*’s work demonstrated that CXCR6 was found to be localised in stroma cells of decidua, especially in the cytoplasm and cell membrane (Fan *et al.* 2018). In our study, we found that CXCL16 is mainly secreted by trophoblast cells and interacts with its receptor CXCR6 in DSCs. Moreover, the protein expression of CXCL16 was significantly lower in pregnant tissue from patients who had experienced spontaneous abortion compared to pregnancy termination for nonmedical reasons. It suggested that CXCL16 might be critical for the initiation and/or maintenance of successful pregnancy. An alternative explanation is that lower CXCL16 levels may be a consequence of spontaneous abortion. To clarify the potential role of CXCL16 in the successful pregnancy, we carry out the following *in vitro* experiments. Due to the significance of decidualization during pregnancy, we tend to explore the role of CXCL16 in the induction of decidualization. *In vitro* decidualised ESCs treated by rhCXCL16 could produce significantly higher PRL and IGFBP-1 than control samples. Anti-CXCL16 administration could reverse the IGFBP-1 and PRL production induced by trophoblast supernatant in decidualised ESCs. Therefore, we conjectured that the lower CXCL16 in pregnancy tissues could lead to the poor decidualization of endometrium, as well as spontaneous abortion. The hypothesis was coinciding with the finding that CXCL16 levels were decreased in the spontaneous abortion tissues compared to pregnancy termination for nonmedical reasons. However, the confirmation of our hypothesis is worthy of further investigation *in vivo*.

Furthermore, we demonstrated that IGFBP-1 and PRL induced by trophoblast supernatant in decidualised ESCs could only be partly reversed after anti-CXCL16 administration. This means that trophoblast cells could produce multiple cytokines that participate in decidualization. As documented, the factors secreted by trophoblast cells such as profilin 1 could also induce the decidualization of human ESCs (Menkhorst *et al.* 2017) In addition, trophoblasts-derived CXCL12 could promote the invasiveness of trophoblasts and enhances the coordination between trophoblasts and DSCs (Zhou *et al.* 2008). CCL25, CCL8 and CXCL10 secreted by trophoblast cells can also be involved in embryo implantation (Sakumoto *et al.* 2017, Weingrill *et al.* 2018). We conjecture that these cytokines may also affect the process of decidualization. It requires further extended investigation, as well as the interaction of these factors.

The PI3K/PTEN/AKT/mechanistic target of rapamycin (mTOR) signal pathway has been clarified to be involved in the CXCR6/CXCL16 biological axis in the cancer realm. Soluble CXCL16, when bound with CXCR6, induces the activation of PI3K. The activated PI3K converts PtdIinsP2 (phosphatidylinositol P2) into PtdIinsP3, which activates downstream PDK-1. PDK-1 might activate AKT, which might directly activate its downstream key protein mTOR. Serine/threonine kinase, mTOR, might phosphorylate and activate its downstream p70S6K and eIF4E-binding protein 1 (4E-BP1), both of which are key regulators of mRNA translation, including HIF-1α and cyclin D1 (Deng *et al.* 2010). In Wang *et al.*’s study, the phosphorylation peak of AKT in the C4-2B prostate cancer cells occurred at 45 min when stimulated by CXCL16, and the phosphorylation of AKT was reduced at 10 min with decreased CXCR6 expression (Wang *et al.* 2008). It is confirmed that PI3K-AKT signalling pathway is involved in the regulation of decidualization (Cha *et al.* 2012). To explore the downstream signalling pathway of CXCL16 in regulating decidualization, we studied the phosphorylation of PI3K, AKT and the expression of PDK1 and cyclin D1 in rhCXCL16-treated decidualised ESCs. We found that rhCXCL16 induced the phosphorylation of PI3K and AKT, and the downstream, PDK1 and cyclin D1, was elevated after 10 to 60 min of incubation. AKT inhibitor-GSK 690693 could inhibit the secretion of PRL by decidualised ESCs. These results verified the efficient effect of rhCXCL16 on PI3K-AKT signalling pathway in decidualised ESCs. However, Akt inhibitor GSK690693 could not affect IGFBP-1 production in rhCXCL16-treated decidualised ESCs. The potential reason is that, through PI3K/AKT pathway, CXCR6/CXCL16 promotes the decidualization of ESCs mainly by PRL production, but not IGFBP-1.

**Figure 7:**
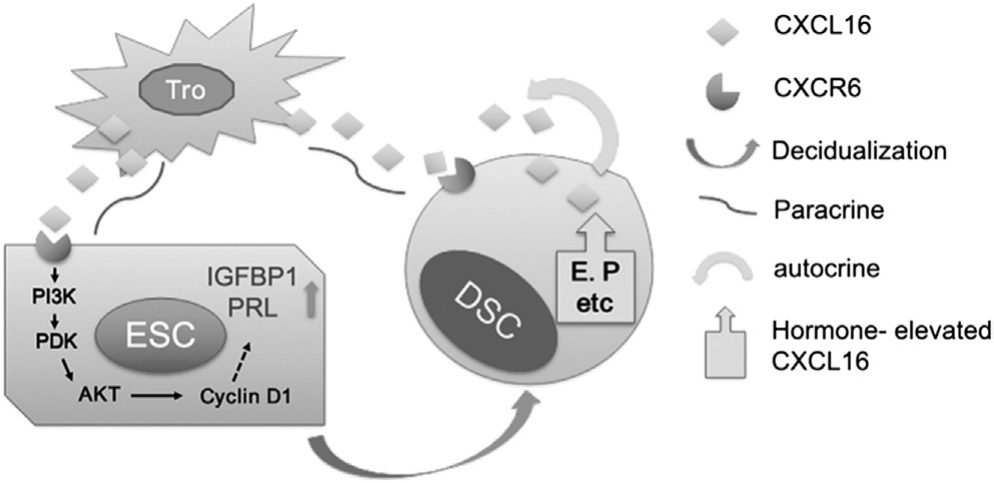
CXCL16/CXCR6 interaction promotes endometrial decidualization via PI3K-PDK1-AKT-Cyclin D1 pathway. The trophoblast-derived CXCL16 interacts with CXCR6 in ESCs, and thus, induces the decidualization of ESCs by activating the PI3K/PDK/AKT/Cyclin D1 signalling pathway. Furthermore, the decidualised ESCs (DSCs) autocrine CXCL16 and steroid hormones (such as oestrogen and progesterone) have positive feedback on DSCs and further improve decidualization during pregnancy.

These results suggested that CXCL16/CXCR6 was elevated in maternal–foetal interface during decidualization. As shown in Fig. 7, our study is the first to demonstrate the mechanism by which CXCL16 regulates the decidualization process. We have verified that CXCL16 is mainly secreted by trophoblast cells, and interacts with its receptor, CXCR6, to initiate decidualization by activating PI3K/PDK1/AKT/Cyclin D1 pathway. Furthermore, the decidualised ESCs (DSCs) autocrine CXCL16 and steroid hormones (such as oestrogen and progesterone) have a positive feedback on DSCs and further improve the decidualization during pregnancy. Additional studies could be conducted to test the efficacy of CXCL16 in promoting the decidualization *in vivo*. The integral results of the regulatory role of CXCL16/CXCR6 axis may represent a novel therapeutic strategy to reduce the chance of recurrent spontaneous abortion or preeclampsia due to impaired decidualization.

## Declaration of interest

The authors declare that there is no conflict of interest that could be perceived as prejudicing the impartiality of the research reported.

## Funding

The present study was supported by the National Natural Science Foundation of China (grant no. 81601354), the National Science Foundation of Jiangsu Province, China (grant no. BK20160128), the Fundamental Research Funds for the Central Universities (grant no. 021414380180), the Open Project Program of Shanghai Key Laboratory of Female Reproductive Endocrine-Related Diseases (grant no. 14DZ2271700) (all to J M), the National Natural Science Foundation of China (grant no. 31671200, 81471513 and 91542108), the Development Fund of Shanghai Talents (grant no. 201557), the Shanghai Rising-Star Program (grant no. 16QA1400800) and the Innovation-oriented Science and Technology Grant from NPFPC Key Laboratory of Reproduction Regulation (CX2017-2) (all to M-Q L), the National Natural Science Foundation of China (grant no. 81601278), the National Science Foundation of Jiangsu Province, China (grant no. BK20160109) (all to Q Z).
